# Quality Assurance of PBPK Modeling Platforms and Guidance on Building, Evaluating, Verifying and Applying PBPK Models Prudently under the Umbrella of Qualification: Why, When, What, How and By Whom?

**DOI:** 10.1007/s11095-022-03250-w

**Published:** 2022-04-20

**Authors:** Sebastian Frechen, Amin Rostami-Hodjegan

**Affiliations:** 1 Bayer AG, Pharmaceuticals, Research & Development, Systems Pharmacology & Medicine, Leverkusen, 51368 Germany; 2 Centre for Applied Pharmacokinetic Research, University of Manchester, Manchester, UK; 3 Certara UK Limited (Simcyp Division), Sheffield, UK

**Keywords:** quality assurance, model-informed drug development, modeling & simulation, physiologically based pharmacokinetic modeling (PBPK), qualification, system pharmacology

## Abstract

Modeling and simulation emerges as a fundamental asset of drug development. Mechanistic modeling builds upon its strength to integrate various data to represent a detailed structural knowledge of a physiological and biological system and is capable of informing numerous drug development and regulatory decisions *via* extrapolations outside clinically studied scenarios. Herein, physiologically based pharmacokinetic (PBPK) modeling is the fastest growing branch, and its use for particular applications is already expected or explicitly recommended by regulatory agencies. Therefore, appropriate applications of PBPK necessitates trust in the predictive capability of the tool, the underlying software platform, and related models. That has triggered a discussion on concepts of ensuring credibility of model-based derived conclusions. Questions like ‘why’, ‘when’, ‘what’, ‘how’ and ‘by whom’ remain open. We seek for harmonization of recent ideas, perceptions, and related terminology. First, we provide an overview on quality assurance of PBPK platforms with the two following concepts. Platform validation: ensuring software integrity, security, traceability, correctness of mathematical models and accuracy of algorithms. Platform qualification: demonstrating the predictive capability of a PBPK platform within a particular context of use. Second, we provide guidance on executing dedicated PBPK studies. A step-by-step framework focuses on the definition of the question of interest, the context of use, the assessment of impact and risk, the definition of the modeling strategy, the evaluation of the platform, performing model development including model building, evaluation and verification, the evaluation of applicability to address the question, and the model application under the umbrella of a qualified platform.

## Introduction

The use of modeling and simulation (M&S) is becoming an integral part of drug discovery and development within the leading pharmaceutical companies although its applications are not uniform and many smaller biotech companies do not have dedicated resources to conduct necessary M&S activities internally considering the narrower stream of their pipelines. Nonetheless, ease of access to dedicated M&S tools and expertise either in the form of internal teams in larger pharmaceutical companies or consultancy services available to smaller companies has provided a base for faster growth of M&S in recent years. Mechanistic modeling is now an established part of M&S as an addition to historical pharmaco-statistical data analysis (1). The growth trajectory of mechanistic models indicates a several fold faster uptake than the increase in more classical M&S activities (2). Classical (mostly but not entirely) empirical models usually depend on a single set or limited sets of observed data from a single clinical study or sets of related studies. Their key role is in the derivation of statistically robust identification and characterization of dose-exposure–response relationships or linking dosage regimens to likely exposure profiles in the context of the study setting. In contrast, mechanistic models integrate a plethora of data sets of often unrelated studies through complex analysis of every element to arrive at a detailed structural knowledge of physiological and biological systems. Such models are often contained by the term ‘systems pharmacology’ (3, 4). With the focus on the mechanistic interplay between pharmacology and the underlying system, such models usually target the prediction in terms of a ‘forward projection’ of experimentally ‘untested’ (as well as ‘untestable’) scenarios. Such predictions might even be relevant to enable therapy for wider sets of patient populations and clinical settings than those common to the original drug development path. The narrow focus of clinical trials and its consequences has been discussed in recent regulatory guidance on widening the diversity of patient populations (5) without declaring the fact that such action may require more intense use of mechanistic models. Obviously, there is no real dichotomy between classical and mechanistic models. In the middle grey zone, one can shift towards the other. Moreover, they are not incompatible with each other and can borrow strength from each other. This has been discussed previously (6) alongside all the confusions that their different philosophy brings up (7), and interested readers can follow such references and other similar publications.

Physiologically based pharmacokinetic (PBPK) modeling is by far the fastest growing branch amongst mechanistic models (2). PBPK applications are now frequent in guiding decision-making during drug development, and they provide substantial support for prescription drug labeling and submissions in a regulatory context (1, 8–11). PBPK provides a unique mechanistic and versatile framework that integrates drug properties and system-specific organism properties and thus allows multiple applications in particular in the area of drug-drug interaction (DDI) risk assessment, exposure prediction in a specific target population, and absorption modeling (12). In this context, PBPK modeling is usually carried out with specialized and established PBPK software platforms such as GastroPlus® (www.simulations-plus.com), PK-Sim® (www.open-systems-pharmacology.org) or Simcyp™ (www.simcyp.com). Although there seems to be distinction between the research-oriented applications of PBPK versus the drug development applications[2], the story of rapid regulatory adoption to answer key questions and the success cases on applications supporting regulatory submissions to various health authorities [8, 10, 13] has created an environment where the use of PBPK for particular application purposes under certain conditions is already almost expected or even explicitly recommended by regulatory agencies (e.g., the European Medicines Agency (EMA) (14) or the US Food and Drug Administration (15)).

**Fig. 1:**
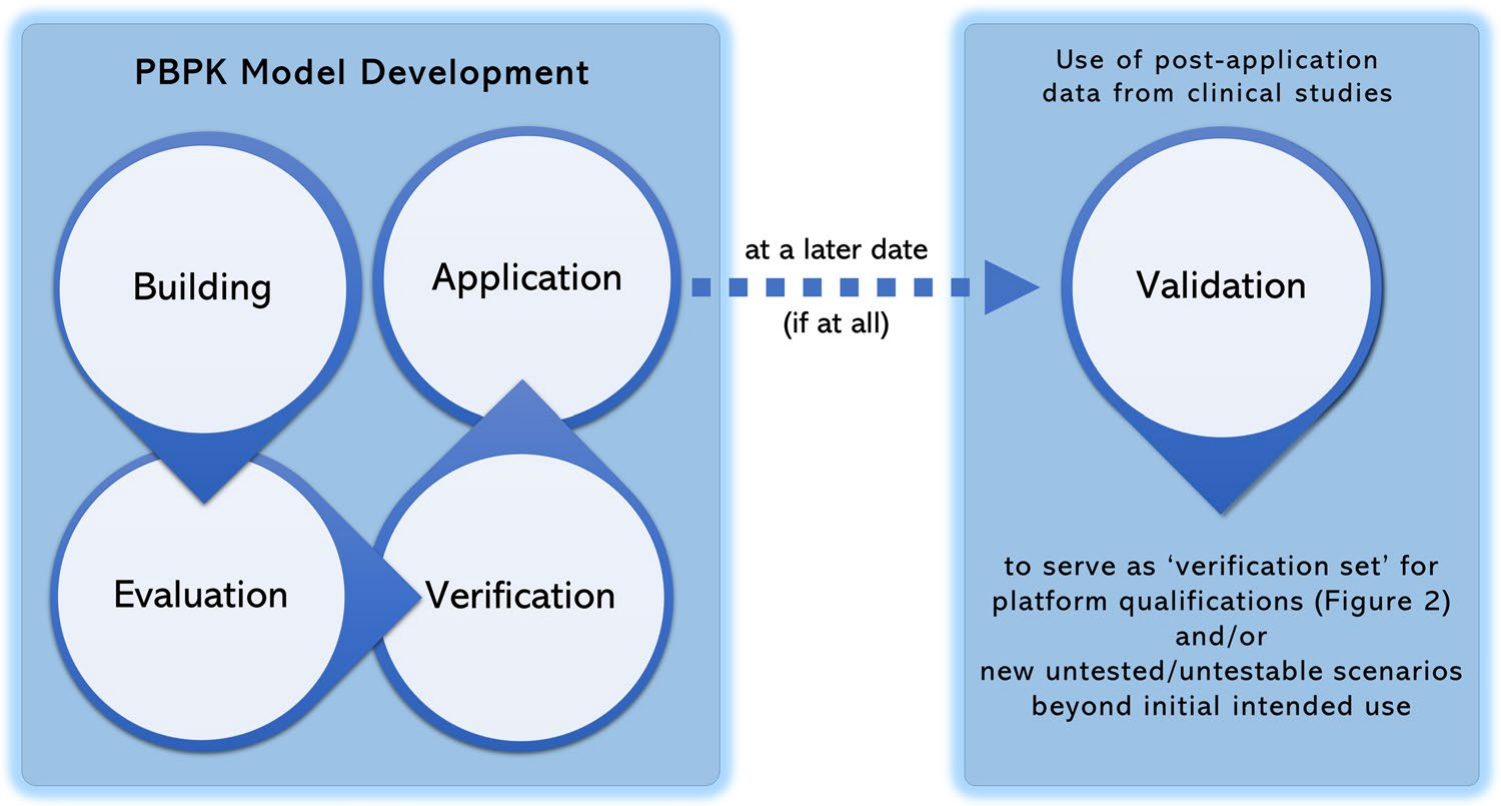
The diagram shows the over-arching strategy in PBPK modeling and its applications. A question of interest and its context of use define the inherent stages of model development. For model building and evaluation, the modeler should consider the balance of ‘bottom-up’ and ‘top-down’ modeling techniques, availability and feasibility of previously established cases and platforms, and apply best practices at every stage. If a model can adequately be verified considering the key property of the model relevant for the intended use, it can be applied. In many cases applications cover conditions, which are untested or untestable at that given time. Hence, ‘validation’ comes at much later stages (if at all) to be of any practical benefit for the intended purpose. However, this can be used as ‘verification set’ for future scenarios. Details on the specific stages of model development are outlined in the respective sections of the section PBPK Modeling Analysis.

As stated earlier, any inference from PBPK modeling applications does not rely on a single specific set of observed data but rather on a scientifically well-founded mechanistic integration of all relevant available knowledge using implicit and explicit assumptions. As a direct consequence, any PBPK modeling application needs to be embedded into a rigorous qualification concept to put the credibility of its derived conclusions into a quantitative perspective (16, 17). But what does qualification mean? Actually, this is comparable to any professional service that is offered in the society and regulated by sets of standards. Imagine a practicing physician offering medical advice and services. Such practice requires a qualification in terms of the license to operate based on passing certain exams, which are governed by an authorized and capable organization or governmental body. Also imagine that the qualification is time sensitive and requires some updates indicating that the previously qualified doctor has considered the advancements of medical knowledge through taking up some credit units related to specific areas. Lastly, imagine that the license to practice can be revoked if the doctor makes repeated major mistakes or misuse, e.g., due to negligence or even criminal intent. This scenario has parallels in applications of PBPK modeling in a qualified environment which is no more than distinguishing the qualified doctor with the license to operate from other people in relation to the fitness to offer medical services. There is a growing body of literature and guidelines that proposes valuable concepts for a qualification framework necessary for PBPK analyses including aspects of verification of PBPK models (7, 11, 12, 16, 17). However, a consensus not only in the concrete implementation but also in the terminology used for certain aspects of such a framework is lacking and may be difficult to reach in foreseeable future considering the challenges of a global synchronization in general. We herein do not intend to invent or propose just another new concept for such a framework but rather would like to follow up on the call for harmonization in the recent white paper by Kuemmel *et al.* (11) and offer our opinion on requirements on the one hand of quality assurance of PBPK platforms and on the other hand of performing qualified PBPK modeling activities. Thus, the primary objective is to complement and reflect on current ideas. There are two principal sections of the document on the over-arching strategy in PBPK modeling (Fig. 1). The first part focuses on general quality assurance of PBPK modeling platforms leading to platform qualifications for particular use cases in general. The target audience for this section mainly would be suppliers of PBPK platforms and regular sponsors of PBPK studies. The second section provides guidance on prerequisites and elements of specific PBPK modeling analyses under the umbrella of platform qualification *via* a step-by-step framework. The main target audience for this part are the regular sponsors of PBPK studies as well as scientists running such modeling studies. Though our focus is PBPK modeling, the presented ideas and concepts are mostly also applicable (or at least translatable) for mechanistic modeling in general.

## PBPK Platform

**Fig. 2:**
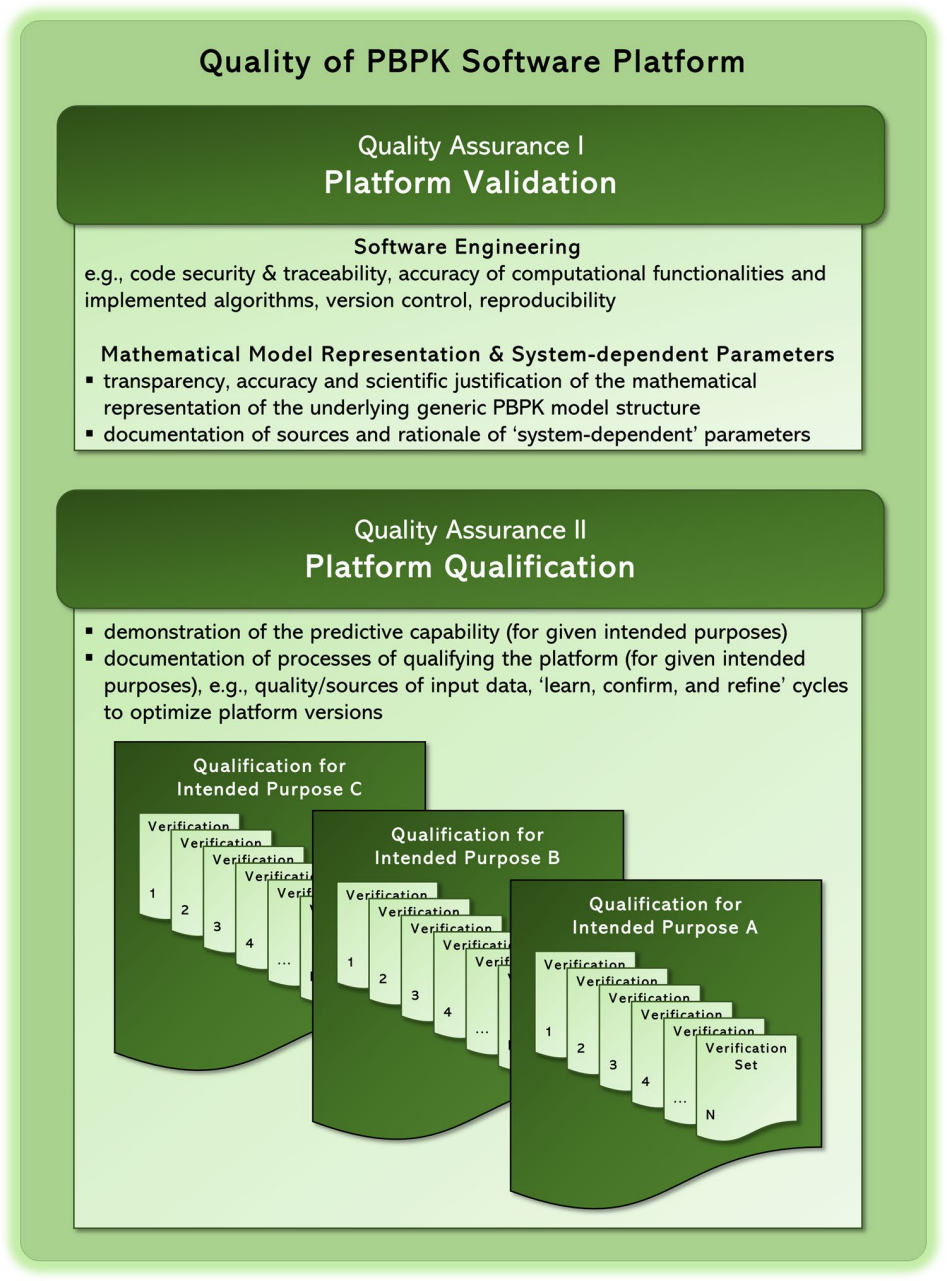
Overview on the quality assurance framework of PBPK software platforms. Suppliers of dedicated specialized PBPK platforms should provide a battery of quality assurance documents to demonstrate platform validation and a series of potential platform qualifications for intended purposes. Modelers using such platforms are responsible to ensure that the potential platform qualification is appropriate within a dedicated analysis. Modelers are responsible for platform qualification on its own only if the intended use is a novel application for which the platform provider has no qualification documents. Details are presented in the section PBPK Platform

A PBPK platform is a software environment that enables the development of PBPK models and their applications. A PBPK model is a mathematical model that allows to simulate the pharmacokinetics of drugs on the basis of an interaction of physiological, physicochemical and biochemical properties and determinants (16). PBPK platforms comprise two major key pillars: (1) a technical computational infrastructure with the provisioning of a generic physiological model framework and (2) a spectrum of potential applications within a specific context of use. While the first key component of a PBPK platform sets up on the program source code with the provision of a runtime environment and graphical user interfaces, it also defines the mathematical equations of the generic PBPK model structure and the corresponding interplay of the so called *system-dependent* and *drug-dependent* parameters (18–20). Hereby, system-dependent parameters describe the typical physiology of human or preclinical species for a reference individual (no variability is assumed) or population (where interindividual variability of each parameter is also considered stochastically). Drug-dependent parameters reflect the physicochemical properties of a compound being considered relevant for its absorption, distribution, metabolism and excretion (ADME) characteristics (21). The second key component of a PBPK platform represents the specific platform’s content in terms of a compilation of libraries, such as compound libraries, specific population libraries etc., and its application potential within a well-defined context of use. In the light of these two major components of a PBPK platform, we herein propose in the following sections corresponding concepts and requirements of quality assurance for PBPK platform suppliers and vendors. Quality assurance with regard to the first pillar is herein defined as ‘*platform validation’* and covers primarily measures and prerequisites to ensure software integrity, security, traceability, code correctness of underlying mathematical models and accuracy of implemented algorithms. Details are outlined below in section **Quality Assurance I: Platform Validation**. Quality assurance with regard to the second pillar is herein defined as ‘*platform qualification’* and covers primarily a concept for the demonstration of the (version-specific) predictive capability of the PBPK platform for a particular context of use (qualification scenarios). Details are outlined below in section **Quality Assurance II: Platform Qualification**. A summary on the concepts is presented in Fig. 2.

### Quality Assurance I: Platform Validation

Suppliers of PBPK platforms should perform version-specific quality assurance measures to ensure the provisioning of a reliable and robust software platform that validate its general purpose of PBPK modeling. A corresponding transparent and comprehensive design and specification documentation, which describes how the platform is developed and maintained, needs to be provided to stakeholders to gain the necessary trust. Importantly, a comprehensive assessment of platform quality should not be restricted to those who have created the software platform. Potential assessors certainly include regulatory agencies but also industrial and academic users under provisions that respect the intellectual property and copyrights in relation to re-engineering for purpose of creating competing products or sharing with direct competitors. Key features and elements of such a quality assurance framework and its documentation should focus on the implementation of (11, 12, 22, 23):

- a proper **software engineering**, i.e., application of a secure source code and release management including version controlling and full traceability, correct implementation of computational functionalities, provision of quality-controlled software installation
- an accurate **mathematical representation** of the underlying generic PBPK model structure and its **system-dependent parameters** (and any variants of the models when there is more than a single option for selection by the users)

#### Software Engineering

The implementation of a proper software engineering backbone is intended to ensure the correctness of implemented model code, so that the software does what it is intended to do from a computational perspective (22). Validation activities include assessments on the accuracy and reliability of the underlying algorithms, solvers and other computational functionalities. In particular, for every newly released feature or platform version, this should be accomplished by code quality analysis (e.g., static code analysis, test coverage), automated standardized batch testing using a comprehensive library of well-defined test cases/models evaluating the behavior of software modules, and peer code reviews. An overview of validation steps and reports should be documented and available for inspection with every version of the platform. Modularity of platform may allow partial tests related to certain modules if changes do evidently not affect any elements outside that module.

A clear and transparent release management should be implemented. This includes planning of the next version release and offering full traceability by means of release notes and a release history listing any changes made in a particular release in comparison to its previous version. Therefore, an appropriate and secure version control and source code management system is required that offers access control, bug tracking, feature requests and task management. It should be noted that for open-source code platforms, special security requirements do apply in addition. Source code modifications, with intention or inadvertently, must not be integrated into the software automatically and without notification. A well-established procedure proposes an iterative approach overseen by the supplier of the open-source software. Source code modifications are proposed and subject to a thorough (technical and scientific) review by the managing supplier of the respective committee or working parties on that open-source platform. A proposal may then be declined or considered for formal integration. In case of a positive decision, re-validation of the software with the integrated proposed changes should be performed and only in case of a successful re-validation, the proposed changes can be accepted as part of an official release. While the source-code is available for modifications by a large group of researchers, this procedure ensures that the official and quality assured versions of the platform are not affected by individual variations in the code, which have not gone through quality assurance. Closed-source software may not suffer from this potential adversity; however, the ability in revising and improving the implemented algorithms through contributions by the research community (outside the software vendor) is severely restricted.

Finally, with the distribution of the platform to the user, it is necessary to warrant reproducibility of the behavior of the software. The issue of reproducibility has been subject of several recent data analyses of models in the space of systems biology (24) with indications that these may not be satisfactory without all the quality assurance steps outlined above. Therefore, a tool for installation validation should be provided by platform suppliers that ensures that the software works fully as intended when installed in the computing environment. Key functionalities should be tested automatically through comparison of reference simulation outputs with locally generated outputs for a set of predefined test simulations.

#### Mathematical Model Representation & System-dependent Parameters

Validation activities on the mathematical model representation focus on determining that a mathematical model implementation accurately represents the conceptual description of the model and its solution from the perspective of the intended use of PBPK modeling (11, 25, 26). PBPK platforms provide predefined generic mathematical models with representations of a system of organs, tissues, the biological interaction with physicochemical and biochemical properties of drugs which allows to simulate whole-body pharmacokinetics of the respective drug, i.e., disintegration and dissolution processes, permeation through barriers and absorption kinetics into the systemic circulation, circulation in blood stream and distribution in tissues, and finally its metabolism and excretion (ADME). Such generic models should be developed based on most commonly accepted and tested scientific principles and assumptions (note that in many cases there might be alternative models within a given platform that can be selected by the user). If then a technically correct implementation and functionality of the physiological framework and related equations that describe the underlying system can transparently be provided to any potential assessor, in particular to authorities, who are independent assessors without any conflict regarding commercial breach of intellectual properties, the generic model structure can be considered validated. Usually, the generic model structure is linked to databases with physiological data representing the physiology of human or preclinical species (system-dependent parameters) as a minimum for typical values and in many cases with all the attributes defining the variability in a given set of the desired target population, whether rooted in genetic or environmental causes. These parameters should be clearly documented and justified to complete the platform validation activities (16, 17). System-dependent parameters may also be subject to qualification measures (see below **Quality Assurance II: Platform Qualification**) when they are key for a specific PBPK application. A prominent example for the latter case is the human ontogeny of a certain enzyme.

The overall validation process can obviously be supported by supplying a comprehensive track record of peer-reviewed publications demonstrating the overall quality of the platform in the light of its general intended use of PBPK modeling.

### Quality Assurance II: Platform Qualification

While platform validation focuses rather on the technical and general quality assurance in the view of the *generic* intended use (i.e., PBPK modeling), platform qualification is defined as a version-specific evaluation to demonstrate the platform’s predictive capability and reliability for a *specific* intended context of use (12, 27). Thus, platform qualification emerges as a concept that ensures the permission to handle the intended use with the application of a specific version of a particular platform (22). The level of confidence for a specific context of use is determined by setting up a qualification scenario comprising a set of closely related simulations to the application case (so called verification sets), the output of which is compared against an external qualification dataset (of observed clinical data). This concept can be extended to run also new application cases with prudence where prior performance may only be deduced from other qualification scenarios, which are not directly related to such a new application case.

The case of ‘predicting CYP3A4-mediated DDI in a typical healthy volunteer population for a new drug’, or the case of ‘translating pharmacokinetics (PK) from adults to neonates and young pediatric populations below 2-years-old for drugs cleared mainly *via* CYP3A4’ would be typical examples of PBPK applications. A respective qualification scenario for the first case would for example be a setup of a CYP3A4-DDI PBPK model network of typical CYP3A4 perpetrators and sensitive CYP3A4 victim drugs and compare simulations against a comprehensive dataset of published clinical DDI studies. For the latter case, a set of simulations of various CYP3A4 substrates in appropriate pediatric age classes compared against published corresponding clinical data could represent a proper qualification scenario. Although anecdotal discussions on number of independent verification sets required for a potential qualification has been floating around from 3 cases, as a minimum, to 10 cases, as a conservative measure, there is no consensus or official position taken by any regulatory agency on the matter of how large and diverse a particular qualification dataset has to be to suffice acceptance of a platform qualification. In the context of such qualification scenarios and the assessment of their performance, the idea of visual predictive checks (VPC) becomes a philosophically different matter than commonly used within classical modeling. The VPC in classical modeling is not a true ‘prediction’ but rather checks the consistency of the model outcome against the same set of data (or a subsection of it) that were used to develop the model itself (7). PBPK qualification scenarios use VPC not for an intended prediction case itself, where ‘no observed data’ are available, but rather for sets of closely related observed datasets external to model building.

Here, it must be highlighted that within a particular PBPK modeling activity, a specific context of use and the related platform qualification is always directly linked to a well-defined question of interest that should be addressed by a PBPK analysis (see section below **PBPK Modeling Analysis**). This implies for PBPK studies, in particular those which target a forward projection, that it is the responsibility of the user or sponsor to ensure that the platform is sufficiently qualified for the intended context of use (11). This includes a rigorous assessment that the current modeling case shows robust similarity in nature with regard to the modeling approach and kind of input data of a serving qualification scenario (see section below **Evaluate Platform Qualification** as subsection of **PBPK Modeling Analysis**). Now it becomes clear why a qualification needs to be assessed on a case-by-case decision (16). Success of a PBPK application (and related submission) depends on the one hand on the specifics of the intended use with its data quality and availability and its resulting breadth and depth of the simulations, and on the other hand on its impact and risk of the particular PBPK study against the alternatives including remaining silent from a modeling perspective, when there is no clinical study data available and/or no possibility to conduct clinical studies within a reasonable timeline (see section below **Assess Impact & Risk** as subsection of **PBPK Modeling Analysis**).

Still, the effort needed to supply a respective *potential* platform qualification along with a PBPK study may overstrain the costs and capabilities for the user or sponsor for such an individual PBPK analysis. Consequently, and at least in areas where common applications are established, it is highly recommended that suppliers of PBPK platforms provide potential platform qualifications of their software system beyond platform validation (see section above **Quality Assurance I: Platform Validation**) for a variety of common intended purposes of PBPK applications, accompanied by respective documentation in terms of qualification reports for respective stakeholders and particularly regulatory authorities. Such qualification reports should enable an efficient assessment of the current predictive performance for existing observations using prespecified metrics and charts of the specific version of the platform for respective intended purposes. This again requires the establishment of an agile and sustainable framework for automated PBPK platform (re-) qualification for platform suppliers. Such a framework should feature the generation of comprehensive and transparent standardized qualification reports to facilitate efficient review for all stakeholders to assess the respective level of confidence, enable *via* automated workflows efficient re-qualification (e.g., for upcoming new releases) and the facilitation of version comparisons, enable versatile and continuous development of qualification scenarios (i.e., extensions by new data or models, tailoring, etc.) and provide full traceability and transparency of all required input files and data (27). It is important to note that such version-specific qualification reports are essential to comply with regulatory requirements (16), since only referencing striking journal publications addressing the specific context of use may just reflect a snapshot in time in terms of a temporary qualification of version of the respective PBPK platform at the time of the publication (27), and thus can only be considered supporting documents. Here, we would like to refer to the above mentioned scenario of a medical doctor with the need for continuous education in the case of keeping up a qualification for professional services such as practicing medicine.

Obviously, suppliers of platforms cannot provide preconfigured potential platform qualifications for each and every specific context of use. Thus, the technical framework for qualification should on the one hand ideally be generic to potentially serve a broad spectrum of intended purposes and with that also be available for and applicable by the users. This again requires the provisioning of technical how-to manuals to enable the development of sustainable qualification scenarios by users. Prominent examples on such qualification frameworks have recently been established and rolled out (27–29).

Every platform qualification scenario requires the use of PBPK compound models. Therefore, platform qualification measures involve model evaluation and verification for the library compounds in use with regard to the context of use. Providing transparent documentation of the particular modeling strategy, model development, input parameters, model features and model performance for each compound model is mandatory (16). In recent history, there have been successful application cases in which such formal platform qualifications leveraged the regulatory acceptance of PBPK studies to address a particular question of interest (30–32).

## PBPK Modeling Analysis

A PBPK analysis is an M&S exercise to address a particular clinical question of interest. In pharmaceutical industry for example, it may facilitate decision-making during drug discovery and development based on available data on a specific drug and wider knowledge about the physiology and biology. This can be in support of trial design as well as informing prescription drug labeling beyond what clinical studies at the time can address. The following section should provide a guidance for modelers on building, evaluation, verification, and application of PBPK models under the umbrella of qualification as part of such a particular exercise to comply with regulatory requirements and integrity in scientific standards. In the narrower sense, PBPK model in this section refers to the drug PBPK model to be established and applied for the explicit (investigational) drug of interest. As mentioned above, we do not intend to invent or define a completely new set of ideas on guidance but rather try to harmonize already established concepts. Hereby, we particularly draw on the recently published FDA white paper by Kuemmel *et al.* (11).

### Define Question of Interest

The first task is to specify the question of interest that should be addressed by the upcoming modeling and simulation PBPK analysis. The question of interest emerges from broader issues of the respective development program of a particular investigational drug. The question defines objectives of the analysis and may range from describing available evidence of clinical data up to fully generate new evidence. Examples for an investigational drug, which is primarily metabolized by CYP3A4, are ‘which labeling prescription information is appropriate for the investigational drug under co-administration of CYP3A4 modulators?’ or ‘what is an appropriate dose that matches the desired exposure known from adults in a pediatric population aged 0 to 2 years?’ If multiple questions should be addressed, the guidance provided in the next section applies in general separately for each individual question of interest. Obviously, overlap may exist, and it is the sponsor’s responsibility to structure the total modeling & simulation PBPK package appropriately.

### Define Context of Use & Specific Intended Purpose

The second task is to define the specific intended purpose and the context of use of the PBPK modeling analysis emerging from the question of interest. The context of use represents the general application area of the specific intended purpose. Given our examples of questions of interest given above, we would for example deal with an analysis targeting an application purpose of ‘predicting CYP3A4-mediated DDI in a typical healthy volunteer population’ or ‘translating PK from adults to a pediatric population aged 0 to 2 years for drugs cleared mainly *via* CYP3A4’. The specific intended purpose of a PBPK analysis defines the exact role and scope of the model on how the question of interest will be addressed. In our two cases this could be for example ‘the effect of the index CYP3A4 modulators A, B, …, and G on the PK of the investigational drug will be simulated to predict and evaluate its DDI liability’ or ‘simulations of the PK of the investigational drug in virtual pediatric populations aged 0 to 2 years will determine an appropriate pediatric dose for this age class’. The specification of the intended purpose should then be completed by defining respective concrete simulation output metrics (e.g., area under the plasma concentration–time curve ratios (AUCR), peak plasma concentration ratios (C_max_R), etc.) that will later allow the development of a quantitative (e.g., a particular dose recommendation) or qualitative (e.g., contraindicated: yes or no) answer to the question of interest in the broader context of the development program.

### Assess Impact & Risk

Before initiating modeling activities, the sponsor of the PBPK study should clearly define the impact of the analysis within the development program and the associated risk in case the model and its simulation results would lead to an incorrect characterization of the efficacy and/or safety profile of the investigational drug.

Historically, modeling and simulations were suggested to be classified into three categories based on their impact depending on the purpose of the analysis, e.g. to either describe, or to justify and support, or to really replace (clinical) evidence, and the weight of model simulation results in decision making: low, medium and high (1, 33). Briefly, low impact analyses are considered descriptive analyses with very limited impact on decision-making for the overall development program (e.g., a PBPK analysis simply to gain a more mechanistic understanding of observations); medium impact analyses provide supportive evidence and contribute to decision-making along with clinical data; high impact analyses provide new evidence in the absence of respective clinical data and their results contribute exclusively to decision-making (e.g., PBPK studies in lieu of clinical studies to inform prescription labeling). The assessment on impact is directly associated with the setup of the modeling strategy, in particular with qualification and verification measures required to finally evaluate the applicability of the analysis to address the question of interest. In line with the EMA guideline on the reporting of PBPK modeling and simulation (16), we propose, for a high impact analysis, a rigorous qualification comprising an adequate number of external verification cases (minimum of 3 cases) as the necessary requirement for acceptance. As part of this package, the provision or generation of a respective associated qualification report for the intended context of use is paramount. However, for any moderate to low impact analysis, the qualification and its included number of external verification cases might be lowered, and in some cases, the use of an internal dataset might be permitted for the analysis until more relevant clinical data become available.

Kuemmel *et al.* (11) proposed to assess independently on the one hand the impact of the analysis and on the other hand the ‘decision consequence’ of a potential incorrect M&S analysis result. Again, three categories are given: for analyses with a low/medium/high decision consequence incorrect decision would result in no/minor to moderate/severe adverse outcomes in patient safety or efficacy. While we highly appreciate and embrace this concept, we would prefer to use the term ‘risk assessment’ for this rating. Also, we propose to simplify the assessment and differentiate only two categories: (1) no substantial risk, and (2) substantial risk that an incorrect result would lead to potential adverse outcomes in patient safety or efficacy. It is also important to consider the risks associated with remaining silent and not performing the analysis (1), in particular when the question of interest is unlikely to be answered with any clinical data in foreseeable future. Such considerations are often ignored for the simple reason that it makes everyone’s job easier. Remaining silent from the modeling perspective due to high model uncertainty but not being able to address the question of interest otherwise actually provokes a situation where the outcome of a treatment would be even more ‘uncertain’ for the group of patients which is affected by that scenario and subject of that unlabeled use due to particular void of information in the label. The high level of off-label drug use in children and all associated consequences (34) are the mere results of the false premise of ‘safer option of leaving a void in the label’ compared to a ‘model-derived recommendation on dose’. Similar scenarios are discussed in the case of severe renal or hepatic impairment (35).

The outcome of this risk assessment again is directly linked to the setup of the M&S strategy to finally evaluate the applicability of the analysis to address the question of interest. While the impact assessment is rather focusing on the question which qualification and verification measures are necessary, the risk assessment should rather be reflected in the required quantitative level of confidence and accuracy of the simulation results. In the case of an identified substantial risk that an incorrect result would lead to potential adverse outcomes, stringent and tailored acceptance criteria need to be derived. Obviously, particular attention needs to be devoted firstly to the dose-exposure–response relationship from an efficacy and/or safety point of view and secondly to the totality of evidence of data around the question of interest. In case no substantial risk is identified, widely used common acceptance limits such as the ‘two-fold’ criterion or the criterion proposed by Guest *et al.* (36) might be sufficient.

Back to our examples: (1) The DDI prediction analysis would be ranked as high impact analysis since clinical studies are replaced by *in silico* studies. Let’s assume that the investigational drug has a broad therapeutic window with a well-defined exposure–response relationship and that a clinical study with a very strong CYP3A4 inhibitor showed a fourfold increase in AUCR of this investigational drug. This allows to assign no substantial risk that an incorrect result would lead to potential adverse outcomes in patient safety or efficacy. Consequently, an extensive qualification is necessary but standard acceptance criteria are probably sufficient. (2) The pediatric dose prediction could be ranked as moderate impact analysis as the dose just serves to inform the study design and the final pediatric dose will determined based on the results of the pediatric clinical trial. Also, the risk is rather not substantial as all pediatric patients will be monitored closely during trial, such that doses may be adjusted based on individual patient responses. Consequently, the standards for a qualification are lower and standard acceptance criteria are also sufficient.

### Define Modeling Strategy

The modeling strategy should outline the specific approach of the PBPK analysis to address the question of interest. Before referring to specific modeling activities around the investigational drug of interest, the evaluation of the respective PBPK platform to be used needs to be specified with regard to platform validation and potential platform qualification (see section above **PBPK Platform**) for the context of use. Then, the first prerequisite is a thorough understanding and definition of the key biological and physiological mechanisms relevant to the question of interest and the specific intended purpose to be represented in the model of the investigational drug. Peters and Dolgos (12) presented an interesting example of a model that is intended for the prediction of food effect: The herein mentioned compound is substrate of CYP3A and P-gp substrate, thus, to finally predict the food effect it is important to gain a quantitative understanding of the relative contributions of absorption, intestinal efflux, intestinal metabolism and hepatic metabolism.

Based on this, the *model development* process starts with listing all input source data required to establish the PBPK model with its key properties relevant for the intended use, e.g., physicochemical data, *in vitro* data and clinical data. The next step is to define its inherent stages:

1. *model building*, i.e., which parameters can be informed from *in vitro* or *in silico* experiments, and which parameters are unknown and could be informed/optimized from selected clinical data?
2. *model evaluation*, i.e., how to assess the general performance of the model with regard to describing the PK of the compound itself and the plausibility of the mechanistic representation of its properties?
3. *model verification* (if applicable), i.e., how to assess (1) whether the key property/properties of the model relevant for the intended use is well reflected in the model and (2) the performance of the model with regard to the specific intended purpose? These questions obviously require appropriate related (model-building independent) comparative clinical data. For our case example ‘predicting CYP3A4-mediated DDI’, a clinical DDI study with a strong CYP3A4 inhibitor could be used as a verification of the model performance with regard to the specific intended purpose of predicting the DDI liability with other CYP3A4 modulators. However, if such data are not available for the intended prediction scenario (which is a common scenario for many genuine forward projections – otherwise ‘why do we need the predictor’!), one can only try to assess the first question *via* data from outside the context of use of the current analysis and needs to infer answers to the second question from a suitable platform qualification with other compounds within the context of use that helps to qualify the actual exercise. That is for example true for our second example of ‘predicting a pediatric dose of a sensitive CYP3A4 substrate’. Obviously, no direct pediatric data are available at that stage. However, a key component of the model is (similar as in the DDI example) the fraction metabolized *via* CYP3A4 of the overall clearance which could be verified *via* the aforementioned DDI study with a strong CYP3A4 inhibitor.
4. *model application*, i.e., which (prospective) simulations are necessary to optimally answer the question of interest?

It must be noted that clinical data may either be used for model development (training data) or for model evaluation and verification (test data). Ideally, this is rather defined upfront, however, refinements during model development following the principle of the ‘learn, confirm, and refine’ paradigm might be considered (37, 38). Note that in case of refinements at the stage of model verification, strictly speaking a ‘genuine’ verification may not be possible anymore. In our opinion, this is not a stop criterion, but implications should be reflected during evaluation of the overall model applicability.

The requirements for accuracy and the level of confidence or the selection rationale of acceptance criteria of the model itself and the related platform for the intended purpose are defined dependent on the type of application and under consideration of the impact and risk of the analysis. This process of appropriately mapping impact and risk to specific analysis requirements may necessitate a team of multidisciplinary experts from various functions being involved in the development program of the compound (11). It should be noted at this point that a reasonable modeling strategy may not always be to finally arrive at delivering the believed most accurate simulation but rather to explore various scenarios (e.g., best-case and worst-case) and evaluate the range of outcomes with regard to the question of interest (39).

### Evaluate Platform

As mentioned above, PBPK models are most frequently built using available specialized platforms. It is the responsibility of the sponsor of a PBPK study to demonstrate the appropriate level of technical and scientific quality assurance: platform validation and platform qualification.

#### Evaluate Platform Validation

Ideally, sufficient and transparent documentation and material is provided by the supplier of the platform to allow the sponsor to simply confirm that required software quality assurance measures have been performed for the specific version of the platform in use. Details on platform validation and respective quality measures are discussed in the section above (**Quality Assurance I: Platform Validation**).

#### Evaluate Platform Qualification

The sponsor of the PBPK study needs to assess the qualification status of the platform given the pursued context of use of the planned PBPK analysis. If the supplier of the platform already has provided a respective (version-specific) potential platform qualification, it is important to evaluate if the scope of this existing potential qualification sufficiently covers the intended use of the PBPK study. This necessitates an assessment of consistency of the specific current case and the qualification set with respect to for example properties of the compound, patient library, (handling of) input (*in vitro*) data, surrounding assumption, etc. If the evaluation is negative, a qualification tailored to the intended application of the model and available data must be carried out by the sponsor. Such a new potential qualification for a platform could then become an integral part of the platform in future (‘open science’ approach). Thus, as discussed above, the provision of a respective technical qualification framework by the platform supplier is essential. If the evaluation is positive, and the model is developed in a platform that is already sufficiently qualified for the intended purpose, no additional activities are required at this stage. Details on platform qualification are outlined in the section above (**Quality Assurance II: Platform Qualification**).

### Model Building

This section should give a high-level overview on key aspects which should be considered for building a PBPK model. Usually, for an investigational drug a base model for healthy volunteers is first developed using experimentally determined or *in silico* predicted physicochemical data, *in vitro* and a set of clinical training data informing absorption, distribution, metabolism, and excretion processes. As the PBPK model is developed to handle a specific task, it must be focused right from the beginning to understand and implement the mechanisms relevant to the question of interest being addressed (12).

While on the one hand quantitative *in vitro–in vivo* extrapolation (IVIVE) has been one of the key driver for the PBPK success story (19), a firm understanding of extracting robust *in vitro* input data and their correct implementation in PBPK models is critical and may also be challenging. *In vitro* data may even be incompatible, in particular as direct inputs for PBPK models. Thus, IVIVE-PBPK workflows may be complex. Harwood *et al.* present an illustrative overview on challenges that exist in obtaining robust *in vitro* parameter estimates, their downstream consequences in a PBPK model and potential solutions in the area of transporter IVIVE (40).

Typically, (final) PBPK models are not built solely by using a ‘bottom-up’ approach, i.e. informing all parameters – especially the drug-related parameters – from *in vitro* or* in silico* experiments. Additionally, model parameters are informed based on observed clinical data (‘top-down’ approach) *via* different available parameter optimization techniques (ranging from rather simple up to complex multidimensional hierarchical statistical approaches) (6). Hereby, parameter non‑identifiability allowing for a proper characterization of the relevant underlying mechanisms. Nonetheless, identifiability in the context of mechanistic models often involves simultaneous model fitting to several sets of observational studies rather than a single case as demonstrated previously (41). A profound statistical quantification of the overall model uncertainty, particularly in the absence of robust systems data, might emerge as the highest barrier and propagation thereof needs to be considered for model application.

During model development the most relevant model assumptions and their justification must be listed. Similarly, model limitations need to be outlined. We recommend applying published or widely accepted (platform-specific) best-practices for model development (42). Additionally, there is plenty of literature giving an overview on specific parts of model development (43–53).

### Model Evaluation

As specified above, model evaluation comprises activities around the assessment of the general performance and mechanistic plausibility of the model with regard to describing the pharmacokinetics of the compound itself, e.g., does the model describe the observed data at various doses and/or under various conditions from healthy volunteers? The key diagnostic tools are visual predictive checks in order to compare model-simulated concentration–time profiles with clinical data and goodness of fit analyses (e.g., residuals over time, residuals against predictions, etc.) and comprehensive precision and bias metrics. One important prerequisite of PBPK models here is that all parameters must have biologically reasonable values. Additionally, general sensitivity analyses should quantify sensitive model parameters, in particular key input parameters from *in vitro* and/or parameters identified *via* parameter estimation from clinical data. The results give valuable information on the robustness of the model performance. Still, we would like to emphasize that the typically claimed sensitivity analysis on single parameter scanning without realization of its change with correlated entities within the system but also even more advanced global sensitivity analyses (GSA) may lead to false conclusions (54, 55). There is a distinction between the interaction of model parameters (that all GSA take into account) and the covariation of the model parameters (that not all GSA cover). The latter means changing a certain parameter should always be associated with changes in all correlated parameters in the system. Single parameter scanning as well as typical GSA ignore this.

As noted above, acceptance at this stage with regard to accuracy and precision of the model is highly dependent on the impact and risk of the analysis und consideration of the question of interest. Thus, model development and evaluation might be an iterative, back and forth process of comparing model-simulated and observed data.

### Model Verification

As specified above, model verification comprises activities around an assessment on the key properties of the model of the investigational drug relevant for the intended (predictive) use and (if applicable) its performance with regard to the specific intended purpose using specific comparator data (11). Thus, verification activities need to ensure the accuracy of the model, confirm the validity of key assumptions, and demonstrate the ability of the model to answer the specific questions of interest (17). We have already outlined that for our two case examples, the verification of the key property ‘fraction metabolized *via* CYP3A4’ could be verified if the model appropriately recovers clinical data with CYP3A4 inhibitors. As mentioned above, an iterative approach of ‘learn, confirm and refine’ with a back and forth between model building and verification may degrade verification to model building and implications (e.g., additional uncertainty) should be considered during the proceeding steps.

For our DDI case example, the acceptability at this stage is supported by the direct assessment of the performance of the model within the context of use given the availability of clinical data with CYP3A4 inhibitors. For the pediatric case example, the performance of the model within the context of the intended purpose cannot directly be assessed in the absence of pediatric data.

Verification activities should be accompanied by specific sensitivity analyses on the parameters defining the key properties of the model for the specific intended use to assess model uncertainty in such parameters and respective impact on model outcome.

### Evaluate Model Applicability

Before applying the developed model for prospective predictions to finally answer the question of interest, an overall evaluation of the model applicability should be conducted and discussed within the totality of evidence depending on the intended use, model assumptions, limitations and uncertainty but also therapeutic area, safety, and efficacy factors (1, 17, 22). This includes in particular a consolidation on the one hand of the outcome of the platform evaluation and on the other hand of the evaluation and verification of the model itself. Sponsors of PBPK studies should hereby focus on whether the suggested accuracy (1) of the predictive performance from the platform qualification (see section above **Evaluate Platform Qualification**) with regard to the context of use, (2) of the general model performance with regard to describing the pharmacokinetics of the compound itself (see section above **Model Evaluation**), and (3) of the specific model performance with regard to the context of use (see section above **Model Verification**) is sufficient given consideration to the previously defined model impact and risk (see section above **Assess Impact & Risk**). This includes an assessment on how robust and certain the model results and conclusions would be. If the evaluation reveals sufficient confidence in the overall predictive performance to answer the question of interest, sponsors can proceed to the final step of the analysis; otherwise, gaps should be analyzed, and reiterations should be considered.

### Model Application in a Qualified Environment

Once all proceeding steps have been successfully completed, the last step is to apply the model under the umbrella of a qualified platform, for example, to provide prospective predictions for a clinically (so far) untested or untestable scenario to finally provide an answer to the question of interest. It is important to mention that scenario-based approaches (best case, worst case, etc.) may also be an option for model application depending on the overall assessment on the applicability (see section above **Evaluation of Model Applicability**).

When for the predictions made at this stage clinical data become available later, a comparison should be initiated (which may be defined as ‘validation’). Then, the case may serve as a verification set and become an integral part of a qualification of the platform to support next forward projections and perpetuate the ‘learn, confirm, and refine’ approach from a platform and PBPK community perspective.

## Summary

**Fig. 3:**
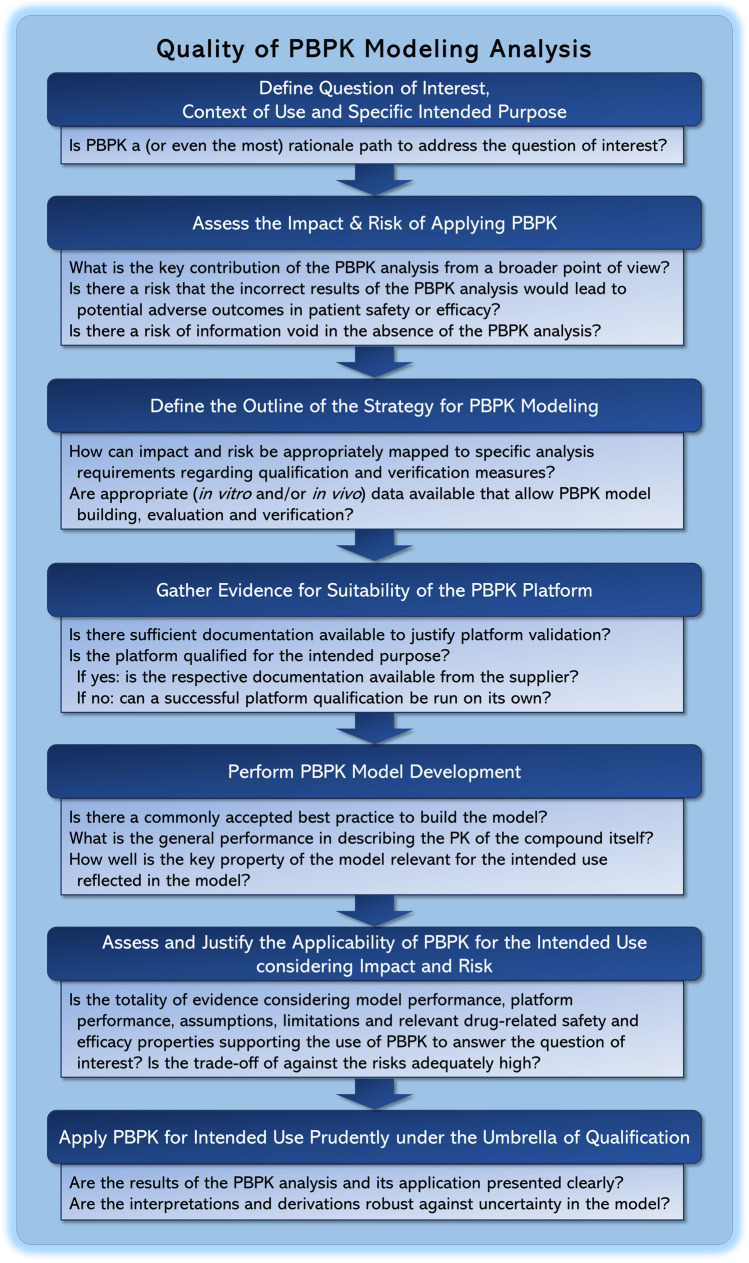
Questions on how to assess quality of a PBPK modeling analysis. Assessing the quality of a PBPK modeling analysis is a long process and involves several steps. Some elements of quality assessment can be facilitated if the software platform has adequate qualification for the intended use of the PBPK model. However, if the platform does not contain such qualifications (e.g., a novel application of the platform) or the platform is not a purpose-built environment for the PBPK modeling, then the modeler has the extra burden of creating such qualifications. The questions are closely linked to the step-by-step framework for dedicated PBPK modeling analyses from defining a question of interest to arrive at the application of PBPK modeling under the umbrella of qualification presented in the section PBPK Modeling Analysis

The use of modeling and simulation is becoming an integral part of drug discovery and development and increasingly receives regulatory support. Mechanistic modeling approaches have their unique strength in informing a variety of drug development and regulatory decisions *via* extrapolations outside the (so far) clinically studied scenarios or populations. Herein, PBPK modeling has shown to be the fastest growing discipline and its prospective predictions are nowadays considered an indispensable source of evidence. Due to its success, it has become a ‘regular thing’ in daily pharmaceutical business. However, appropriate application of PBPK within a context of use to answer a particular question of interest necessitates trust in the predictive capability of the tool itself, the underlying software platform, and related models. This has triggered an ongoing debate on concepts such as platform qualification, model verifications, etc., but for many aspects no consensus on questions like ‘why’, ‘when’, ‘what’, ‘how’ and ‘by whom’ has been reached yet. We herein seek for harmonization of recent ideas and perceptions on these questions and propose a unified terminology. First, we have provided the reasoning for qualification frameworks and offered our opinion on quality assurance requirements for specialized PBPK platforms including the presentation of a concept of ‘platform validation’, i.e., to warrant software integrity, security, traceability, code correctness of underlying mathematical models and accuracy of implemented algorithms, and a concept of ‘platform qualification’, i.e., to demonstrate the predictive capability of a PBPK platform within a particular context of use. Secondly, we have provided guidance for modelers on relevant aspects of a dedicated PBPK study by providing a theoretical step-by-step framework based on the recently published whitepaper by Kuemmel *et al.* (11). It targets to arrive in the last step at the application of a PBPK model under the umbrella of a qualified platform. We focus on (1) the definition of the question of interest, (2) the context of use and the specific intended purpose, (3) the assessment of impact and risk of the analysis, (4) the definition of the modeling strategy, (5) the evaluation of the platform to be used, key aspects of model development including (6) model building, (7) model evaluation and (8) model verification, (9) the evaluation of the overall model applicability to address the question of interest, and (10) lastly on the final task of model application. Associated key questions assessing quality of a dedicated analysis are outlined in Fig. 3.

It should be noted that we have virtually avoided the term ‘model validation’ within this framework. In our opinion, model validation could be understood as validation of an originally forward prediction in terms of the model did what it was intended for or what it should have done (7). Thus, such validation may always only happen after the event when (and if at all) clinical data for the predicted condition become available. In reality, the most useful stage of the use of PBPK is when such data is not available. Therefore, in this context, the term is rather useless. Of course, in the case that data become available, such a validation can be used as a verification set for a potential platform qualification within a forward prediction of another subsequent respective PBPK study or of a scenario, for which no direct clinical data exist.

We advocate for the adoption of the herein presented concepts and ideas with the intention to contribute to more standardization firstly in quality assurance of PBPK platforms and secondly in running PBPK studies and a harmonized terminology in this area. Though we have set a clear focus on PBPK modeling, we believe that in principle these concepts and ideas do also apply for mechanistic modeling in general.
